# (*E*)-5-[(1,5-Dimethyl-3-oxo-2-phenyl-2,3-dihydro-1*H*-pyrazol-4-yl)imino­meth­yl]-2-meth­oxy­phenyl 4-chloro­benzene­sulfonate

**DOI:** 10.1107/S160053681202627X

**Published:** 2012-06-16

**Authors:** Tian-Xiang Lei

**Affiliations:** a Department Of Electrical Engineering, North China Electric Power University, Baoding City, Hebei Province 071003, People’s Republic of China

## Abstract

In the title compound, C_25_H_22_ClN_3_O_5_S, the two N atoms in the pyrazole ring have a pyramidal environment, with the sums of the valence angles around them being 349.3 (2) and 357.5 (2)°. The phenyl ring is twisted by 50.97 (12)° from the pyrazole mean plane. In the crystal, pairs of weak C—H⋯O hydrogen bonds link the mol­ecules into inversion dimers.

## Related literature

For general background to the use of Schiff base derivatives in the development of protein and enzyme mimics, see: Santos *et al.* (2001 ▶). For related structures, see: Zhang *et al.* (2006 ▶); Han *et al.* (2008 ▶); Guo *et al.* (2010 ▶). For reference bond-length data, see: Allen *et al.* (1987 ▶).
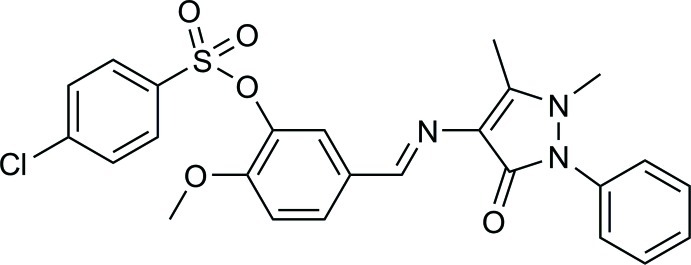


## Experimental

### 

#### Crystal data

C_25_H_22_ClN_3_O_5_S
*M* *_r_* = 511.98
Monoclinic, 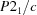

*a* = 11.063 (2) Å
*b* = 10.153 (2) Å
*c* = 22.159 (4) Å
β = 98.73 (3)°
*V* = 2460.1 (8) Å^3^
*Z* = 4
Mo *K*α radiation
μ = 0.28 mm^−1^
*T* = 294 K
0.30 × 0.26 × 0.18 mm

#### Data collection

Bruker SMART APEX CCD area-detector diffractometer
Absorption correction: multi-scan (*SADABS*; Sheldrick, 1996 ▶) *T*_min_ = 0.907, *T*_max_ = 0.951
19683 measured reflections
4315 independent reflections
3016 reflections with *I* > 2σ(*I*)
*R*_int_ = 0.052

#### Refinement

*R*[*F* ^2^ > 2σ(*F* ^2^)] = 0.056
*wR*(*F* ^2^) = 0.154
*S* = 1.00
4315 reflections
319 parameters
H-atom parameters constrained
Δρ_max_ = 0.34 e Å^−3^
Δρ_min_ = −0.45 e Å^−3^

### 

Data collection: *SMART* (Bruker, 1999 ▶); cell refinement: *SAINT* (Bruker, 1999 ▶); data reduction: *SAINT*; program(s) used to solve structure: *SHELXS97* (Sheldrick, 2008 ▶); program(s) used to refine structure: *SHELXL97* (Sheldrick, 2008 ▶); molecular graphics: *SHELXTL* (Sheldrick, 2008 ▶); software used to prepare material for publication: *SHELXTL*.

## Supplementary Material

Crystal structure: contains datablock(s) I, global. DOI: 10.1107/S160053681202627X/cv5310sup1.cif

Structure factors: contains datablock(s) I. DOI: 10.1107/S160053681202627X/cv5310Isup2.hkl

Supplementary material file. DOI: 10.1107/S160053681202627X/cv5310Isup3.cml

Additional supplementary materials:  crystallographic information; 3D view; checkCIF report

## Figures and Tables

**Table 1:** Hydrogen-bond geometry (Å, °)

*D*—H⋯*A*	*D*—H	H⋯*A*	*D*⋯*A*	*D*—H⋯*A*
C11—H11⋯O5^i^	0.93	2.43	3.199 (3)	140

Symmetry code: (i) 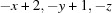.

**Fig. 1.**
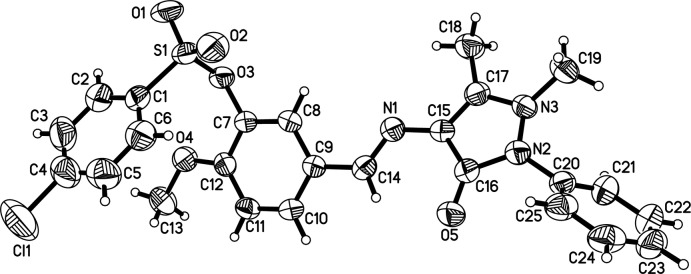
The structure of the title molecule, with displacement ellipsoids for non-H atoms drawn at the 50% probability level.

**Fig. 2.**
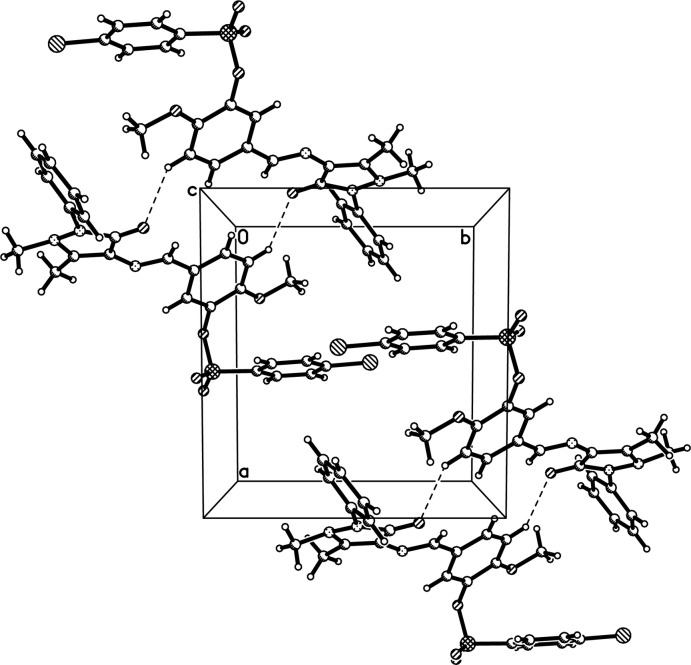
A portion of the crystal packing showing weak C—H···O interactions as dashed lines.

## supplementary crystallographic information

### Comment

Schiff bases have extensively been studied because of their potentially biological activities such as protein and enzyme mimics (Santos *et al.*, 2001). Among the large number of compounds, 4-amino-1,5-dimethyl-2-phenylpyrazol-3-one forms a variety of Schiff bases with aldehydes, and the synthesis and crystal structures of some of them, such as (*E*)-5-((1,5-Dimethyl-3-oxo-2-phenyl-2,3-dihydro-1*H*-pyrazol-4-ylimino)methyl) -2-methoxyphenyl 4-bromobenzenesulfonate (Guo *et al.*, 2010), (*E*)-4-((1,5-Dimethyl-3-oxo-2-phenyl-2,3-dihydro-1*H*-pyrazol-4-ylimino)methyl) phenyl 4-bromobenzenesulfonate (Han *et al.*, 2008) and (*E*)-4-(2-(4-Chlorobenzyloxy)benzylideneamino) -2,3-dimethyl-1-phenyl-1,2-dihydropyrazol-5-one (Zhang *et al.*, 2006) have been reported. Herewith we report the synthesis and crystal structure of the title Schiff base compound.

In the title molecule (Fig. 1), bond lengths and angles are within normal ranges (Allen *et al.*, 1987). Two N atoms in the pyrazole ring have a pyramidal environment with the sums of the valence angles around them of 349.3 (2) and 357.5 (2)°, respectively. The phenyl ring is twisted at 50.97 (12)° from the pyrazole mean plane. The central benzene ring (C7—C12) with three attached atoms (C14/O3/O4) is nearly planar, with an r.m.s. deviation for fitted atoms of 0.0392 Å. The mean plane of this fragment formss dihedral angles of 32.89 (8)°, 38.18 (10)° and 82.42 (7)°, respectively, with the the pyrazolone ring (C15—C17/N1—N3/O5), the chlorobenzene ring (C1—C6) and the terminal phenyl ring (C20—C25). Similar values of 32.02 (14)°, 37.49 (18)° and 80.52 (13)°, respectively, were observed in isostructural (*E*)-5-((1,5-Dimethyl-3-oxo-2-phenyl-2,3-dihydro-1*H*-pyrazol -4-ylimino)methyl)-2-methoxyphenyl 4-bromobenzenesulfonate (Guo *et al.*, 2010).

In the crystal, non-classical intermolecular C11—H11···O5═ C16 hydrogen bonds (Table 1) form inversion-related dimers (Fig. 2).

### Experimental

An anhydrous ethanol solution (100 ml) of 5-formyl-2-methoxyphenyl 4-chlorobenzenesulfonate (3.27 g, 10 mmol) was added to an anhydrous ethanol solution (100 ml) of 4-amino-1,5-dimethyl-2-phenylpyrazol-3-one (2.03 g, 10 mmol) and the mixture refluxed for 3 h under N_2_, giving a yellow precipitate. The product was isolated, recrystallized from acetonitrile, and then dried in a vacuum to give pure compound (I) in 76% yield. Yellow single crystals of the title compound suitable for X-ray analysis were obtained by slow evaporation of an acetonitrile solution.

### Refinement

The H atoms were included in calculated positions and refined using a riding model approximation. Constrained C—H bond lengths and isotropic U parameters: 0.93 Å and *U*_iso_(H) = 1.2*U*_eq_(C) for C*sp*^2^—H; 0.96 Å and *U*_iso_(H) = 1.5*U*_eq_(C) for methyl C—H.

### Crystal data

**Table:**

C_25_H_22_ClN_3_O_5_S	*F*(000) = 1064
*M**_r_* = 511.98	*D*_x_ = 1.382 Mg m^−^^3^
Monoclinic, *P*2_1_/*c*	Mo *K*α radiation, λ = 0.71073 Å
Hall symbol: -P 2ybc	Cell parameters from 5316 reflections
*a* = 11.063 (2) Å	θ = 2.7–27.9°
*b* = 10.153 (2) Å	µ = 0.28 mm^−^^1^
*c* = 22.159 (4) Å	*T* = 294 K
β = 98.73 (3)°	Block, yellow
*V* = 2460.1 (8) Å^3^	0.30 × 0.26 × 0.18 mm
*Z* = 4	

### Data collection

**Table:**

Bruker SMART APEX CCD area-detector diffractometer	4315 independent reflections
Radiation source: fine-focus sealed tube	3016 reflections with *I* > 2σ(*I*)
Graphite monochromator	*R*_int_ = 0.052
φ and ω scans	θ_max_ = 25.0°, θ_min_ = 2.4°
Absorption correction: multi-scan (*SADABS*; Sheldrick, 1996)	*h* = −11→13
*T*_min_ = 0.907, *T*_max_ = 0.951	*k* = −12→11
19683 measured reflections	*l* = −26→26

### Refinement

**Table:**

Refinement on *F*^2^	Primary atom site location: structure-invariant direct methods
Least-squares matrix: full	Secondary atom site location: difference Fourier map
*R*[*F*^2^ > 2σ(*F*^2^)] = 0.056	Hydrogen site location: inferred from neighbouring sites
*wR*(*F*^2^) = 0.154	H-atom parameters constrained
*S* = 1.00	*w* = 1/[σ^2^(*F*_o_^2^) + (0.0919*P*)^2^] where *P* = (*F*_o_^2^ + 2*F*_c_^2^)/3
4315 reflections	(Δ/σ)_max_ < 0.001
319 parameters	Δρ_max_ = 0.34 e Å^−^^3^
0 restraints	Δρ_min_ = −0.45 e Å^−^^3^

### Special details

**Table:**

Geometry. All e.s.d.'s (except the e.s.d. in the dihedral angle between two l.s. planes) are estimated using the full covariance matrix. The cell e.s.d.'s are taken into account individually in the estimation of e.s.d.'s in distances, angles and torsion angles; correlations between e.s.d.'s in cell parameters are only used when they are defined by crystal symmetry. An approximate (isotropic) treatment of cell e.s.d.'s is used for estimating e.s.d.'s involving l.s. planes.
Refinement. Refinement of *F*^2^ against ALL reflections. The weighted *R*-factor *wR* and goodness of fit *S* are based on *F*^2^, conventional *R*-factors *R* are based on *F*, with *F* set to zero for negative *F*^2^. The threshold expression of *F*^2^ > 2σ(*F*^2^) is used only for calculating *R*-factors(gt) *etc*. and is not relevant to the choice of reflections for refinement. *R*-factors based on *F*^2^ are statistically about twice as large as those based on *F*, and *R*- factors based on ALL data will be even larger.

### Fractional atomic coordinates and isotropic or equivalent isotropic displacement parameters (Å^2^)

**Table:**

	*x*	*y*	*z*	*U*_iso_*/*U*_eq_	
Cl1	0.47353 (11)	1.05947 (9)	0.12284 (7)	0.1381 (5)	
S1	0.44039 (6)	0.44761 (7)	0.13133 (3)	0.0563 (3)	
N1	0.80799 (17)	0.1811 (2)	0.00538 (9)	0.0454 (5)	
N2	0.91575 (19)	0.0090 (2)	−0.11867 (10)	0.0515 (6)	
N3	0.88868 (18)	−0.09430 (19)	−0.08078 (9)	0.0477 (5)	
O1	0.36954 (18)	0.4061 (2)	0.17629 (10)	0.0786 (7)	
O2	0.41778 (17)	0.3950 (2)	0.07100 (9)	0.0729 (6)	
O3	0.57713 (14)	0.40946 (15)	0.16147 (7)	0.0497 (5)	
O4	0.70559 (15)	0.62701 (17)	0.19204 (8)	0.0548 (5)	
O5	0.92591 (17)	0.23713 (16)	−0.11241 (9)	0.0617 (5)	
C1	0.4426 (2)	0.6207 (3)	0.12761 (12)	0.0516 (7)	
C2	0.4230 (3)	0.6931 (3)	0.17786 (15)	0.0699 (8)	
H2	0.4046	0.6510	0.2126	0.084*	
C3	0.4310 (3)	0.8292 (3)	0.17608 (19)	0.0835 (10)	
H3	0.4171	0.8797	0.2094	0.100*	
C4	0.4600 (3)	0.8884 (3)	0.1241 (2)	0.0829 (10)	
C5	0.4784 (3)	0.8173 (4)	0.07370 (18)	0.0809 (10)	
H5	0.4966	0.8596	0.0389	0.097*	
C6	0.4693 (2)	0.6812 (3)	0.07553 (14)	0.0651 (8)	
H6	0.4812	0.6311	0.0418	0.078*	
C7	0.67271 (19)	0.4424 (2)	0.12844 (10)	0.0406 (6)	
C8	0.7020 (2)	0.3589 (2)	0.08434 (11)	0.0440 (6)	
H8	0.6593	0.2804	0.0761	0.053*	
C9	0.7967 (2)	0.3923 (2)	0.05159 (11)	0.0426 (6)	
C10	0.8574 (2)	0.5106 (2)	0.06549 (11)	0.0480 (6)	
H10	0.9176	0.5362	0.0428	0.058*	
C11	0.8311 (2)	0.5919 (2)	0.11203 (11)	0.0462 (6)	
H11	0.8754	0.6690	0.1213	0.055*	
C12	0.7384 (2)	0.5579 (2)	0.14468 (11)	0.0425 (6)	
C13	0.7683 (3)	0.7483 (3)	0.20859 (15)	0.0705 (9)	
H13A	0.8534	0.7307	0.2222	0.106*	
H13B	0.7334	0.7898	0.2409	0.106*	
H13C	0.7602	0.8058	0.1738	0.106*	
C14	0.8334 (2)	0.3028 (3)	0.00548 (11)	0.0479 (6)	
H14	0.8762	0.3361	−0.0242	0.057*	
C15	0.8452 (2)	0.0964 (2)	−0.03798 (11)	0.0426 (6)	
C16	0.8973 (2)	0.1298 (2)	−0.09185 (11)	0.0464 (6)	
C17	0.8386 (2)	−0.0375 (2)	−0.03448 (11)	0.0454 (6)	
C18	0.7882 (3)	−0.1191 (3)	0.01137 (13)	0.0641 (8)	
H18A	0.7636	−0.0631	0.0422	0.096*	
H18B	0.8497	−0.1794	0.0299	0.096*	
H18C	0.7188	−0.1676	−0.0083	0.096*	
C19	0.8505 (3)	−0.2198 (3)	−0.10901 (15)	0.0740 (9)	
H19A	0.8321	−0.2801	−0.0783	0.111*	
H19B	0.9152	−0.2551	−0.1285	0.111*	
H19C	0.7790	−0.2070	−0.1389	0.111*	
C20	0.9860 (2)	−0.0126 (2)	−0.16676 (11)	0.0464 (6)	
C21	1.0877 (2)	−0.0934 (3)	−0.15858 (13)	0.0590 (7)	
H21	1.1128	−0.1339	−0.1212	0.071*	
C22	1.1517 (3)	−0.1131 (3)	−0.20709 (15)	0.0713 (9)	
H22	1.2197	−0.1681	−0.2022	0.086*	
C23	1.1156 (3)	−0.0521 (3)	−0.26230 (15)	0.0746 (10)	
H23	1.1578	−0.0676	−0.2949	0.090*	
C24	1.0169 (3)	0.0320 (3)	−0.26911 (13)	0.0698 (9)	
H24	0.9948	0.0764	−0.3058	0.084*	
C25	0.9499 (2)	0.0510 (3)	−0.22172 (12)	0.0581 (7)	
H25	0.8817	0.1057	−0.2268	0.070*	

### Atomic displacement parameters (Å^2^)

**Table:**

	*U*^11^	*U*^22^	*U*^33^	*U*^12^	*U*^13^	*U*^23^
Cl1	0.1248 (9)	0.0543 (6)	0.2232 (15)	0.0018 (5)	−0.0119 (9)	0.0116 (7)
S1	0.0484 (4)	0.0558 (5)	0.0687 (5)	−0.0162 (3)	0.0215 (3)	−0.0118 (3)
N1	0.0461 (11)	0.0448 (13)	0.0460 (12)	−0.0022 (10)	0.0092 (9)	−0.0025 (9)
N2	0.0614 (13)	0.0431 (12)	0.0546 (14)	−0.0067 (10)	0.0239 (11)	−0.0020 (10)
N3	0.0568 (12)	0.0386 (12)	0.0500 (12)	−0.0078 (10)	0.0156 (10)	−0.0030 (9)
O1	0.0710 (13)	0.0767 (14)	0.0990 (16)	−0.0271 (11)	0.0480 (12)	−0.0073 (12)
O2	0.0650 (12)	0.0821 (15)	0.0712 (14)	−0.0193 (10)	0.0095 (10)	−0.0306 (11)
O3	0.0537 (10)	0.0459 (10)	0.0535 (11)	−0.0081 (8)	0.0208 (8)	0.0004 (8)
O4	0.0549 (11)	0.0530 (11)	0.0576 (11)	−0.0096 (8)	0.0124 (9)	−0.0172 (8)
O5	0.0811 (13)	0.0442 (11)	0.0669 (13)	−0.0084 (9)	0.0345 (11)	0.0020 (9)
C1	0.0391 (14)	0.0532 (16)	0.0631 (17)	−0.0016 (11)	0.0102 (12)	−0.0045 (13)
C2	0.0656 (18)	0.065 (2)	0.083 (2)	−0.0015 (16)	0.0242 (16)	−0.0099 (17)
C3	0.073 (2)	0.066 (2)	0.111 (3)	0.0068 (17)	0.010 (2)	−0.026 (2)
C4	0.0586 (19)	0.058 (2)	0.126 (3)	0.0053 (16)	−0.006 (2)	0.009 (2)
C5	0.067 (2)	0.073 (2)	0.099 (3)	0.0016 (17)	−0.0010 (19)	0.026 (2)
C6	0.0547 (17)	0.069 (2)	0.069 (2)	0.0006 (14)	−0.0002 (14)	0.0035 (15)
C7	0.0411 (13)	0.0379 (13)	0.0448 (14)	−0.0021 (10)	0.0125 (11)	0.0016 (10)
C8	0.0463 (13)	0.0356 (13)	0.0512 (15)	−0.0052 (11)	0.0111 (11)	0.0004 (11)
C9	0.0453 (13)	0.0372 (13)	0.0469 (14)	−0.0023 (11)	0.0122 (11)	0.0010 (10)
C10	0.0462 (14)	0.0453 (15)	0.0558 (16)	−0.0061 (11)	0.0184 (12)	0.0029 (12)
C11	0.0426 (13)	0.0395 (14)	0.0560 (16)	−0.0081 (11)	0.0061 (11)	−0.0025 (11)
C12	0.0423 (14)	0.0383 (13)	0.0458 (14)	0.0016 (11)	0.0034 (11)	−0.0020 (11)
C13	0.0780 (19)	0.0533 (18)	0.081 (2)	−0.0145 (15)	0.0125 (16)	−0.0280 (15)
C14	0.0472 (14)	0.0502 (16)	0.0491 (15)	−0.0022 (12)	0.0163 (12)	−0.0024 (12)
C15	0.0400 (13)	0.0410 (14)	0.0476 (14)	−0.0054 (11)	0.0095 (11)	−0.0009 (11)
C16	0.0429 (13)	0.0451 (15)	0.0530 (15)	−0.0036 (11)	0.0131 (11)	−0.0041 (12)
C17	0.0429 (14)	0.0480 (15)	0.0462 (15)	−0.0034 (11)	0.0098 (11)	0.0003 (11)
C18	0.080 (2)	0.0533 (17)	0.0636 (19)	−0.0114 (14)	0.0245 (16)	0.0040 (14)
C19	0.094 (2)	0.0513 (18)	0.083 (2)	−0.0229 (16)	0.0352 (19)	−0.0200 (16)
C20	0.0508 (15)	0.0472 (15)	0.0439 (14)	−0.0112 (12)	0.0153 (11)	−0.0061 (11)
C21	0.0636 (17)	0.0621 (18)	0.0544 (17)	0.0006 (14)	0.0187 (14)	−0.0044 (13)
C22	0.072 (2)	0.071 (2)	0.078 (2)	0.0013 (16)	0.0342 (17)	−0.0096 (17)
C23	0.090 (2)	0.080 (2)	0.063 (2)	−0.0271 (19)	0.0385 (18)	−0.0237 (17)
C24	0.089 (2)	0.078 (2)	0.0429 (16)	−0.0328 (19)	0.0130 (15)	−0.0034 (14)
C25	0.0553 (16)	0.0638 (19)	0.0547 (17)	−0.0154 (13)	0.0066 (13)	−0.0030 (14)

### Geometric parameters (Å, º)

**Table:**

Cl1—C4	1.743 (3)	C9—C10	1.388 (3)
S1—O1	1.4218 (18)	C9—C14	1.470 (3)
S1—O2	1.4263 (19)	C10—C11	1.386 (3)
S1—O3	1.6055 (18)	C10—H10	0.9300
S1—C1	1.760 (3)	C11—C12	1.385 (3)
N1—C14	1.267 (3)	C11—H11	0.9300
N1—C15	1.397 (3)	C13—H13A	0.9600
N2—C16	1.391 (3)	C13—H13B	0.9600
N2—N3	1.404 (3)	C13—H13C	0.9600
N2—C20	1.428 (3)	C14—H14	0.9300
N3—C17	1.365 (3)	C15—C17	1.365 (3)
N3—C19	1.453 (3)	C15—C16	1.443 (3)
O3—C7	1.414 (2)	C17—C18	1.484 (3)
O4—C12	1.357 (3)	C18—H18A	0.9600
O4—C13	1.434 (3)	C18—H18B	0.9600
O5—C16	1.241 (3)	C18—H18C	0.9600
C1—C2	1.379 (4)	C19—H19A	0.9600
C1—C6	1.379 (4)	C19—H19B	0.9600
C2—C3	1.386 (4)	C19—H19C	0.9600
C2—H2	0.9300	C20—C21	1.381 (4)
C3—C4	1.379 (5)	C20—C25	1.383 (3)
C3—H3	0.9300	C21—C22	1.389 (3)
C4—C5	1.371 (5)	C21—H21	0.9300
C5—C6	1.386 (5)	C22—C23	1.375 (4)
C5—H5	0.9300	C22—H22	0.9300
C6—H6	0.9300	C23—C24	1.377 (4)
C7—C8	1.370 (3)	C23—H23	0.9300
C7—C12	1.397 (3)	C24—C25	1.388 (4)
C8—C9	1.404 (3)	C24—H24	0.9300
C8—H8	0.9300	C25—H25	0.9300
			
O1—S1—O2	120.58 (13)	C11—C12—C7	118.2 (2)
O1—S1—O3	102.82 (12)	O4—C13—H13A	109.5
O2—S1—O3	108.64 (11)	O4—C13—H13B	109.5
O1—S1—C1	110.01 (12)	H13A—C13—H13B	109.5
O2—S1—C1	109.40 (14)	O4—C13—H13C	109.5
O3—S1—C1	103.96 (10)	H13A—C13—H13C	109.5
C14—N1—C15	120.9 (2)	H13B—C13—H13C	109.5
C16—N2—N3	110.16 (19)	N1—C14—C9	121.3 (2)
C16—N2—C20	125.9 (2)	N1—C14—H14	119.4
N3—N2—C20	121.39 (19)	C9—C14—H14	119.4
C17—N3—N2	106.35 (19)	C17—C15—N1	123.4 (2)
C17—N3—C19	124.7 (2)	C17—C15—C16	108.2 (2)
N2—N3—C19	118.2 (2)	N1—C15—C16	128.3 (2)
C7—O3—S1	117.31 (14)	O5—C16—N2	123.7 (2)
C12—O4—C13	117.80 (19)	O5—C16—C15	131.8 (2)
C2—C1—C6	121.2 (3)	N2—C16—C15	104.4 (2)
C2—C1—S1	119.3 (2)	C15—C17—N3	110.3 (2)
C6—C1—S1	119.4 (2)	C15—C17—C18	128.6 (2)
C1—C2—C3	119.4 (3)	N3—C17—C18	121.1 (2)
C1—C2—H2	120.3	C17—C18—H18A	109.5
C3—C2—H2	120.3	C17—C18—H18B	109.5
C4—C3—C2	118.9 (3)	H18A—C18—H18B	109.5
C4—C3—H3	120.6	C17—C18—H18C	109.5
C2—C3—H3	120.6	H18A—C18—H18C	109.5
C5—C4—C3	122.2 (3)	H18B—C18—H18C	109.5
C5—C4—Cl1	119.1 (3)	N3—C19—H19A	109.5
C3—C4—Cl1	118.7 (3)	N3—C19—H19B	109.5
C4—C5—C6	118.7 (3)	H19A—C19—H19B	109.5
C4—C5—H5	120.6	N3—C19—H19C	109.5
C6—C5—H5	120.6	H19A—C19—H19C	109.5
C1—C6—C5	119.6 (3)	H19B—C19—H19C	109.5
C1—C6—H6	120.2	C21—C20—C25	120.9 (2)
C5—C6—H6	120.2	C21—C20—N2	121.2 (2)
C8—C7—C12	122.3 (2)	C25—C20—N2	117.9 (2)
C8—C7—O3	119.9 (2)	C20—C21—C22	119.0 (3)
C12—C7—O3	117.74 (19)	C20—C21—H21	120.5
C7—C8—C9	119.7 (2)	C22—C21—H21	120.5
C7—C8—H8	120.2	C23—C22—C21	120.7 (3)
C9—C8—H8	120.2	C23—C22—H22	119.7
C10—C9—C8	117.9 (2)	C21—C22—H22	119.7
C10—C9—C14	121.0 (2)	C22—C23—C24	119.7 (3)
C8—C9—C14	121.1 (2)	C22—C23—H23	120.2
C11—C10—C9	122.1 (2)	C24—C23—H23	120.2
C11—C10—H10	118.9	C23—C24—C25	120.6 (3)
C9—C10—H10	118.9	C23—C24—H24	119.7
C12—C11—C10	119.7 (2)	C25—C24—H24	119.7
C12—C11—H11	120.1	C20—C25—C24	119.0 (3)
C10—C11—H11	120.1	C20—C25—H25	120.5
O4—C12—C11	125.5 (2)	C24—C25—H25	120.5
O4—C12—C7	116.35 (19)		

### Hydrogen-bond geometry (Å, º)

**Table:**

*D*—H···*A*	*D*—H	H···*A*	*D*···*A*	*D*—H···*A*
C11—H11···O5^i^	0.93	2.43	3.199 (3)	140

Symmetry code: (i) −*x*+2, −*y*+1, −*z*.
